# Effect of MicroRNA-138 on Tumor Necrosis Factor-Alpha-Induced Suppression of Osteogenic Differentiation of Dental Pulp Stem Cells and Underlying Mechanism

**DOI:** 10.1155/2022/7230167

**Published:** 2022-07-08

**Authors:** Wenzhe Liu, Kankui Wu, Wancui Wu

**Affiliations:** Department of Stomatology, The Second Affiliated Hospital of Guangzhou Medical University, Guangzhou, 510260 Guangdong, China

## Abstract

High doses of tumor necrosis factor-*α* (TNF-*α*) suppress osteogenic differentiation of human dental pulp stem cells (hDPSCs). In the present study, we aimed to explore the role and potential regulatory mechanism of microRNA-138 (miR-138) in the osteogenic differentiation of hDPSCs after treatment with a high dose of TNF-*α*. The hDPSCs were cultured in osteogenic medium with or without 50 ng/ml TNF-*α*. The miR-138 levels were upregulated during osteogenic differentiation of the hDPSCs following TNF-*α* treatment. The miR-138 overexpression accelerated but miR-138 knockdown alleviated the TNF-*α*-induced suppression of the alkaline phosphatase activity, calcium deposition, and protein abundance of dentin sialophosphoprotein, dentin matrix protein 1, bone sialoprotein, and osteopontin during osteogenic differentiation induction of hDPSCs. Additionally, miR-138 overexpression accelerated but miR-138 knockdown alleviated the suppression of the focal adhesion kinase- (FAK-) extracellular signal-regulated kinase 1/2 (ERK1/2) signaling pathway during osteogenic differentiation induction of hDPSCs under TNF-*α* treatment. In conclusion, miR-138 accelerates TNF-*α*-induced suppression of osteogenic differentiation of hDPSCs. Inactivation of the FAK-ERK1/2 signaling pathway may be one of the mechanisms underlying the effect of miR-138. Inhibition of miR-138 expression may be a strategy to weaken the inhibitory effect of high-dose TNF-*α* on the osteogenic differentiation of hDPSCs.

## 1. Introduction

Periodontitis refers to chronic inflammation of periodontal tissue. Its main characteristic is the destruction of periodontal tissues, which ultimately leads to tooth loss [1]. Inflammation affects the recovery of periodontal tissue, specifically the repair of alveolar bone defects [1, 2]. Conventionally, periodontitis is treated with basic treatments and ancillary drugs to control inflammation [3]. However, repair of periodontal bone tissue defects in patients with periodontitis remains a major challenge for oral clinicians. Recently, tissue engineering technology has developed rapidly and has been gradually applied to oral disease research. The combination of growth factors, seed cells, and three-dimensional scaffold materials provides new possibilities for the regeneration of alveolar bone defects. Dental pulp stem cells (DPSCs) are a type of seed cells with strong self-renewal ability and multidirectional differentiation capacity *in vivo* and *in vitro* [4, 5]. Inflammation of periodontal tissue can cause a series of changes that affect the regenerative repair capabilities of DPSCs. The differentiation ability of DPSCs can be enhanced in a weak inflammatory environment but can be weakened in a strong inflammatory environment [6, 7]. Therefore, it is necessary to study the treatment of alveolar bone defects and understand how they contribute to the changes and mechanisms underlying the osteogenic differentiation ability of DPSCs in a strong inflammatory environment.

MicroRNAs (miRNAs) (approximately 20–24 nucleotides) are important post-transcription regulators of gene expression that bind to sequences in the 3′-untranslated region of the target messenger RNA [8]. MicroRNAs play a vital role in regulating osteogenic differentiation of stem cells [9, 10]. Moreover, some miRNAs, such as miR-17, miR-21, and miR-148a, are involved in osteogenic differentiation of stem cells in inflammatory microenvironments [11–13]. In this study, we aimed to investigate the role of miR-138 in the osteogenic differentiation of DPSCs in an inflammatory microenvironment. The role of miR-138 in regulating cancer pathogenesis has been widely studied [14]. In addition, miR-138 is involved in regulating stem cell differentiation, especially osteogenic differentiation [15–18]. miR-138 overexpression is believed to suppress while miR-138 knockdown promotes osteogenic differentiation of stem cells [17, 19]. To date, the role and regulatory mechanisms of miR-138 in osteogenic differentiation of human DPSCs (hDPSCs) have not been reported.

In the present study, we aimed to explore the effect of miR-138 on the osteogenic differentiation of hDPSCs following treatment with tumor necrosis factor-*α* (TNF-*α*), an important proinflammatory cytokine produced during the periodontal inflammatory response that plays a key role in regulating bone formation [20]. Subsequently, we analyzed the effect of miR-138 on osteogenic differentiation of hDPSCs following treatment with TNF-*α* and the underlying potential regulatory mechanism.

## 2. Materials and Methods

### 2.1. Isolation and Identification of hDPSCs

hDPSCs were successfully isolated as described previously [21]. Dental pulp tissues were collected from the decayed teeth of healthy individuals (aged 19–29 years old). Informed consent was obtained from all participants. All experimental protocols were carried out in accordance with the guidelines established by the Declaration of Helsinki and were approved by the Institutional Research Ethics Committee of the Second Affiliated Hospital of Guangzhou Medical University. After washing with 0.01 M sterilized phosphate buffer saline, dental pulp tissues were cut into small pieces (approximately 1.0 × 1.0 × 1.0 mm). After digestion with 0.3% type I collagenase and 0.4% dispase (1 : 1) at 37°C for 1 h, the pulp was filtered through a 100 *μ*m Falcon Cell Strainer to obtain a cell suspension. After centrifugation at 800 rpm for 5 min and washing with 0.01 M phosphate buffer saline, the cell pellet was resuspended in minimal essential medium containing 20% fetal bovine serum and cultured in a humidified atmosphere of 5% CO_2_ at 37°C. Fresh minimal essential medium with 20% fetal bovine serum was replenished every three days.

Cells isolated from the third passage were identified using flow cytometry based on hDPSC surface antigens. The harvested cells were incubated in phosphate-buffered saline containing 0.1% fetal bovine serum with fluorescein-conjugated monoclonal antibodies against CD73, CD90, CD105, CD34, and CD45 (BD Biosciences, San Jose, CA, USA). After incubation for 45 min, flow cytometry was performed using a FACSCalibur flow cytometer (BD Biosciences).

### 2.2. Cell Transfection

The negative miRNA control (miR-NC, 5′-UCACAACCUCCUAGAAAGAGUAGA-3′), miR-138 mimic (5′-AGCUGGUGUUGUGAAUCAGGCCG-3′), and miR-138 inhibitor (5′-CGGCCUGAUUCACAACACCAGCU-3′) were purchased from Suzhou GenePharma (China). hDPSCs were seeded in 6-well plates (2 × 10^5^ cells/well) and cultured overnight at 37°C in a 5% CO_2_ incubator. The next day, oligonucleotide transfection (50 nM for miR-NC and miR-138 mimic; 100 nM for miR-138 inhibitor) was performed using Lipofectamine 2000 (Invitrogen, Carlsbad, CA, USA).

### 2.3. Treatment Protocol of hDPSCs

For subsequent experiments, hDPSCs were seeded in 6-well plates (1 × 10^5^ cells per well). To induce osteogenic differentiation, hDPSCs were cultured in osteogenic medium (minimal essential medium with 10% fetal bovine serum, 100 mmol/L dexamethasone, 10 mM *β*-glycerophosphate, and 0.05 mmol/L ascorbic acid). To investigate the expression of miR-138 in TNF-*α*-treated hDPSCs during the induction of osteogenic differentiation, hDPSCs were cultured in osteogenic medium with or without TNF-*α* (50 ng/mL) for 3, 5, 7, and 14 days. Previous studies have shown that TNF-*α* suppresses the osteogenic differentiation of hDPSCs at high concentrations (50 ng/mL) [7]. Therefore, 50 ng/mL TNF-*α* was chosen to treat hDPSCs and create a strong inflammatory environment *in vitro*. To investigate the effect of miR-138 on TNF-*α*-induced changes during the induction of osteogenic differentiation and the underlying mechanism, hDPSCs were divided into five groups. Cells in the blank group were cultured in osteogenic medium for 7 days, and cells in the TNF-*α* group were cultured in osteogenic medium with TNF-*α* (50 ng/mL) for 7 days. Cells in the miR-NC + TNF-*α* group were pretransfected with miR-NC for 24 h and cultured in osteogenic medium with TNF-*α* (50 ng/mL) for 7 days. Cells in the miR-138 + TNF-*α* group were pretransfected with miR-138 for 24 h and cultured in osteogenic medium with TNF-*α* (50 ng/mL) for 7 days, and the cells in the miR-138 inhibitor + TNF-*α* group were pretransfected with the miR-138 inhibitor for 24 h and cultured in osteogenic medium with TNF-*α* (50 ng/mL) for 7 days. For the abovementioned experiments, the culture medium was changed every 2 days.

### 2.4. Alkaline Phosphatase (ALP) Activity Assay

The ALP activity assay was performed using the total protein of the treated cells according to the instructions of the ALP Activity Kit (Beijing Solarbio Science & Technology Co. Ltd., Beijing, China). The total protein concentration was quantified using the Pierce™ Rapid Gold BCA Protein Assay Kit (Pierce, Rockford, IL, USA). The absorbance was measured at 510 nm. The ALP activity of each microgram of protein was measured according to the total protein concentration. The ALP activity in the blank group was normalized to 1.

### 2.5. Alizarin Red S Staining

After treatment, the hDPSCs cultured on coverslips were fixed with 4% paraformaldehyde for 20 min. After washing with phosphate buffer saline, alizarin red S staining solution was added dropwise to cover the cells and they were stained for 1–5 min. After washing twice with phosphate buffer saline, the cells were observed under a light microscope and photographs were taken. The calcium deposition-positive cells were stained orange-red.

### 2.6. RNA Extraction and Reverse Transcription Quantitative Polymerase Chain Reaction (RT-qPCR) Analysis

Total RNA was extracted from cells using TRIzol (Invitrogen, Carlsbad, CA, USA). One microgram RNA was reverse transcribed using the ImProm-II™ Reverse Transcription System (Promega, Madison, WI, USA). miR-138 expression was detected by qPCR using SYBR Green qPCR SuperMix (Invitrogen), and qPCR was performed on a 7500 Real-Time PCR System (Applied Biosystems). RNA U6 small nuclear 1 (U6) was used as the internal reference. Gene expression was measured in triplicates and quantified using the 2^−ΔΔ*CT*^ method. The reverse transcription primers (5′-3′) for miR-138 and U6 were CTCAACTGGTGTCGTGGAGTCGGCAATTCAGTTGAGCGGCCTGA and AACGCTTCACGAATTTGCGT, respectively. The forward and reverse primers (5′-3′) for the qPCR reaction of miR-138 were ACACTCCAGCTGGGAGCTGGTGTTGT and CTCAACTGGTGTCGTGGA, respectively. The forward and reverse primers (5′-3′) for the qPCR reaction of U6 were CTCGCTTCGGCAGCACA and AACGCTTCACGAATTTGCGT, respectively.

### 2.7. Western Blot Analysis

Total protein isolation from the abovementioned cells and Western blot analysis were performed using the conventional approach. Thirty micrograms of protein was loaded per lane. The dilutions of the primary antibodies were as follows: anti-glyceraldehyde-3-phosphate dehydrogenase (GAPDH) (1 : 5000), anti-TNF-*α* (1 : 800), anti-RUNX family transcription factor 2 (RUNX2) (1 : 1000), anti-dentin sialophosphoprotein (DSPP) (1 : 1500), anti-dentin matrix protein 1 (DMP-1) (1 : 1000), anti-osteopontin (OPN) (1 : 1000), anti-bone sialoprotein (BSP) (1 : 1500), anti-focal adhesion kinase (FAK) (1 : 1000), anti-extracellular signal-regulated kinase (ERK) 1/2 (1 : 1000), and anti-phosphorylated (p)-ERK1/2 (1 : 2000). The secondary antibody used in this study was goat anti-mouse IgG H&L (HRP) at a dilution of 1 : 10000. All the antibodies were purchased from Abcam (Cambridge, MA, USA). The integrated optical density of the protein bands was measured using the Pro-Plus software (version 6.0; Media Cybernetics, Rockville, MD, USA). The integrated optical density ratio of the target protein (except p-ERK1/2) and GAPDH was expressed as relative protein abundance. The relative abundance of p-ERK1/2 was normalized to that of GAPDH and total ERK1/2. The relative protein abundance in the control group was 1.

### 2.8. Statistical Analyses

**Figure 1:**
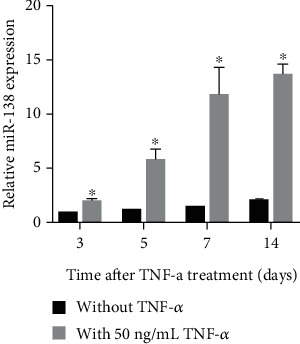
Expression of miR-138 cultured in osteogenic medium with or without treatment with 50 ng/mL TNF-*α* for 3, 5, 7, and 14 days, as detected via RT-qPCR. The bar graphs are means of relative miR-138 expression from three independent experiments. The error bars are standard deviations. ^∗^*P* < 0.05, when compared to the without TNF-*α* group.

The Statistical Package for the Social Sciences (SPSS) version 19.0 software (IBM Inc., Armonk, NY, USA) was used for statistical analyses. After verification using the Shapiro-Wilk test and Levene test, all data in this study conformed to a normal distribution and satisfied the homogeneity of variances. Differences between more than two groups were analyzed using one-way analysis of variance, followed by a post hoc LSD test. The differences between the two groups on the same day in Figure 1 were analyzed using an independent *t*-test. The correlation between TNF-*α* treatment duration and miR-138 expression level was analyzed via linear regression analysis using GraphPad Prism (version 7.0; GraphPad Software, San Diego, CA, USA). Statistical significance was set at *P* < 0.05.

## 3. Results

### 3.1. Isolation and Culture of hDPSCs

**Figure 2:**
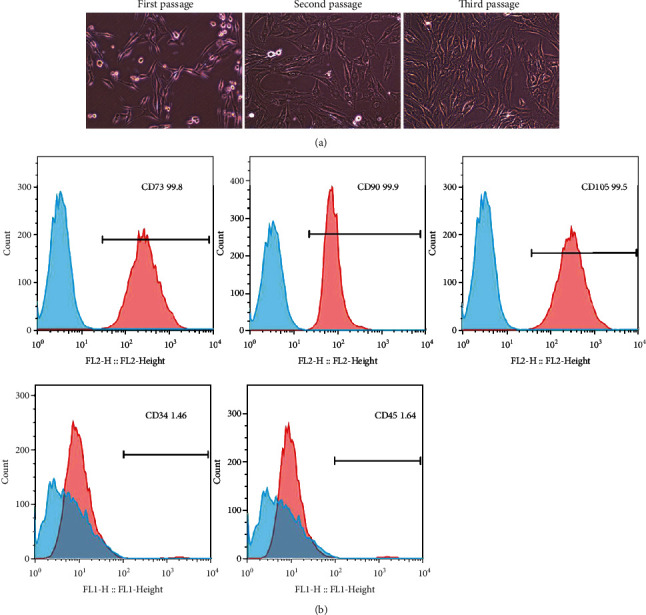
Isolation and culture of hDPSCs. (a) hDPSCs of first passage, second passage, and third passage were observed using an inverted microscope (magnification: 100x). (b) hDPSCs identified via flow cytometry analysis.

Microscopy images showed that hDPSCs from the third passage were long fusiform fibroblast-like or polygonal (Figure 2(a)). Moreover, hDPSCs from the third passage were positive for mesenchymal stem cell surface markers CD90 (99.9%), CD73 (99.8%), and CD105 (99.5%) and lacked the expression of endothelial cell markers CD34 (1.46%) and CD45 (1.64%) (Figure 2(b)).

### 3.2. miR-138 Expression Was Increased during the Osteogenic Differentiation of hDPSCs by TNF-*α*

miR-138 levels were increased in hDPSCs treated with TNF-*α* (50 ng/mL) compared to those in hDPSCs treated without TNF-*α* after treatment for 3, 5, 7, and 14 days (Figure 1), suggesting that TNF-*α* treatment can induce miR-138 expression. Linear regression analysis showed that the increase in miR-138 levels induced by TNF-*α* treatment was not time dependent (*P* = 0.1249).

### 3.3. Effect of miR-138 on the Osteogenic Differentiation of hDPSCs under TNF-*α* Treatment

**Figure 3:**
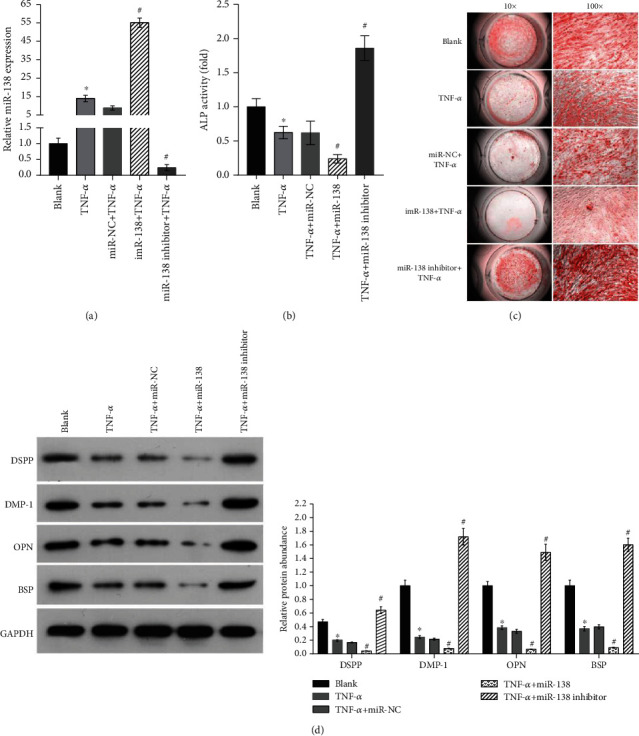
Effect of miR-138 on the osteogenic differentiation of hDPSCs under TNF-*α* treatment. hDPSCs were divided into 5 groups: the blank group (cultured in osteogenic medium), TNF-*α* (cultured in osteogenic medium with 50 ng/mL TNF-*α*), miR-NC + TNF-*α* group (pretransfected with miR-NC for 24 h and cultured in osteogenic medium with 50 ng/mL TNF-*α*), miR-138 + TNF-*α* group (pretransfected with miR-138 for 24 h and cultured in osteogenic medium with 50 ng/mL TNF-*α*), and miR-138 inhibitor + TNF-*α* group (pretransfected with miR-138 inhibitor for 24 h and cultured in osteogenic medium with 50 ng/mL TNF-*α*) and cultured for 7 days. After culturing for 7 days, cells were harvested for RT-qPCR and the detection of miR-138 expression levels (a), ALP activity assay (b), alizarin red S staining (c), and Western blotting to analyze the expression of DSPP, DMP-1, OPN, and BSP (d, e). Representative images of Western blotting (d). Bar graph demonstrating the relative protein levels (e). The bar graphs are means from three independent experiments. The error bars are standard deviations. ^∗^*P* < 0.05, when compared to the blank group; ^#^*P* < 0.05, when compared to the miR-NC + TNF-*α* group.

Because there was a slight change in miR-138 levels from day 7 to 14, treatment with TNF-*α* for 7 days was chosen for the following assays. Next, we analyzed the osteogenic differentiation of hDPSCs in the blank, TNF-*α*, miR-NC + TNF-*α*, miR-138 + TNF-*α*, and miR-138 inhibitor + TNF-*α* groups. RT-qPCR analysis revealed that the miR-138 level was upregulated in the miR-138 + TNF-*α* group, while it was downregulated in the miR-138 inhibitor + TNF-*α* group compared with that in the miR-NC + TNF-*α* group (Figure 3(a)). The ALP activity decreased (Figure 3(b)), calcium deposition was reduced (Figure 3(c)), and the protein abundance of DSPP, DMP-1, OPN, and BSP was decreased (Figure 3(d)) in the TNF-*α* group compared with the values in the blank group. These effects of TNF-*α* treatment were enhanced by miR-138 mimic transfection but were weakened by miR-138 inhibitor transfection (Figure 3). These results indicate that miR-138 overexpression can accelerate the TNF-*α*-induced suppression of osteogenic differentiation of hDPSCs, while miR-138 knockdown can alleviate this effect.

### 3.4. Effect of miR-138 on the FAK-ERK1/2 Signaling Pathway in hDPSCs under TNF-*α* Treatment

**Figure 4:**
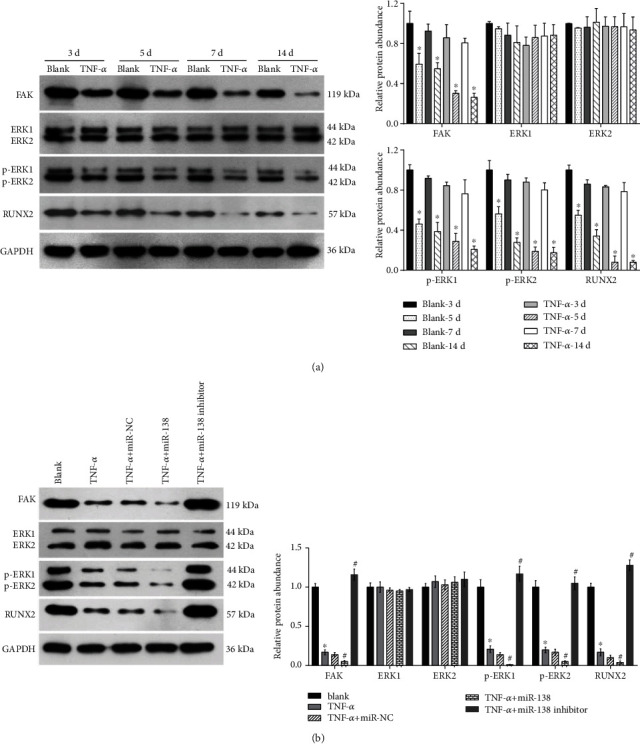
Effect of miR-138 on the FAK-ERK1/2 signaling pathway in hDPSCs under TNF-*α* treatment. (a, b) Protein expression of FAK, ERK1/2, p-ERK1/2, and RUNX2 in hDPSCs cultured in osteogenic medium with or without TNF-*α* (50 ng/mL) for 3, 5, 7, and 14 days. ^∗^*P* < 0.05, TNF-*α* group vs. blank group (same day). (c, d) Protein expression of FAK, ERK1/2, p-ERK1/2, and RUNX2 in the blank group (cultured in osteogenic medium), TNF-*α* group (cultured in osteogenic medium with 50 ng/mL TNF-*α*), miR-NC + TNF-*α* group (pretransfected with miR-NC for 24 h and cultured in osteogenic medium with 50 ng/mL TNF-*α*), miR-138 + TNF-*α* group (pretransfected with miR-138 for 24 h and cultured in osteogenic medium with 50 ng/mL TNF-*α*), and miR-138 inhibitor + TNF-*α* group (pretransfected with miR-138 inhibitor for 24 h and cultured in osteogenic medium with 50 ng/mL TNF-*α*) for 7 days. (a, c) Representative images of Western blotting. (b, d) Bar graph demonstrating the relative protein levels. The bar graphs are means from three independent experiments. The error bars are standard deviations. ^∗^*P* < 0.05, when compared to the blank group; ^#^*P* < 0.05, when compared to the miR-NC + TNF-*α* group.

miR-138 can target FAK during osteogenic differentiation of human stromal stem cells [17]. Hence, we predicted that miR-138 may play a role in regulating the osteogenic differentiation of hDPSCs in an inflammatory microenvironment by targeting FAK and its downstream signaling proteins, ERK1/2 and RUNX2. We found that the protein levels of FAK, p-ERK1/2, and RUNX2 gradually decreased in hDPSCs treated with TNF-*α* compared with those in hDPSCs treated without TNF-*α* (Figure 4(a)). The expression level of ERK1/2 did not change in hDPSCs after TNF-*α* treatment. These results indicate that TNF-*α* treatment can suppress the FAK-ERK1/2 signaling pathway during the osteogenic differentiation of hDPSCs.

We found that the protein abundances of FAK, p-ERK1/2, and RUNX2 were significantly lower in the TNF-*α* group than in the blank group, lower in the miR-138 + TNF-*α* group than in the miR-NC + TNF-*α* group, and higher in the miR-138 inhibitor + TNF-*α* group than in the miR-NC + TNF-*α* group (Figure 4(b)). The expression level of ERK1/2 did not change significantly among the five groups (Figure 4(b)). These results revealed that miR-138 overexpression accelerated but miR-138 knockdown alleviated the suppression of the ERK1/2 signaling pathway in hDPSCs following TNF-*α* treatment.

## 4. Discussion

Periodontitis is an important contributing factor to alveolar bone defects. Members of the IL-1, IL-6, and TNF families are key proinflammatory cytokines involved in periodontitis [22]. Among these families, only the binding between TNF family members and their related receptors plays a role in suppressing osteoblastic activity and inducing osteoclastic activity [22]. TNF-*α* belongs to the TNF/TNFR cytokine superfamily and is a proinflammatory cytokine involved in various biological processes. Liao et al. reported higher TNF-*α* protein levels in periodontal tissues of a rat periodontitis model than those in the control group [23]. TNF-*α* levels were higher in the serum of patients with chronic periodontitis than those in control subjects with minimally inflamed periodontal tissues [24]. Furthermore, TNF-*α* was found at higher levels in both saliva and serum of subjects with periodontitis compared to the levels in healthy subjects [25]. Periodontal therapy can decrease TNF-*α* levels [26]. Moreover, high doses of TNF-*α* have been reported to suppress osteogenic differentiation of hDPSCs, which are key cells in alveolar bone defect repair [7]. These studies suggest that alleviation of TNF-*α*-induced suppression of osteogenic differentiation of hDPSCs may be a way to treat alveolar bone defects induced by periodontitis. Therefore, we focused on studying TNF-*α*-induced suppression of osteogenic differentiation of hDPSCs. In this study, we explored the function and mechanism of miR-138 in osteogenic differentiation of hDPSCs following treatment with high-dose TNF-*α*.

We found that TNF-*α* treatment upregulated miR-138 levels and suppressed osteogenic differentiation of hDPSCs, indicating that miR-138 may play a role in the suppression of osteogenic differentiation induced by TNF-*α* treatment. Our results showed that miR-138 overexpression accelerated the TNF-*α*-induced suppression of osteogenic differentiation of hDPSCs and miR-138 knockdown alleviated this effect. Based on these results, we hypothesized that miR-138 plays a suppressive role in regulating the osteogenic differentiation of hDPSCs following TNF-*α* treatment. In addition, miR-138 overexpression decreased whereas miR-138 knockdown increased the expression of DSPP, DMP-1, OPN, and BSP. DSPP, DMP-1, OPN, and BSP belong to the small integrin-binding ligand, n-linked glycoprotein (SIBLING) family, which plays important developmental regulatory roles in bone tissue formation and tooth development [27]. DSPP and DMP-1 are dentin-specific proteins [28, 29], and OPN and BSP are osteogenic differentiation markers [30]. Therefore, the effect of miR-138 on these proteins further supports our hypothesis. Previous studies have also revealed that miR-138 is involved in osteogenic differentiation of MSCs. Zhang et al. confirmed that miR-138-5p knockdown promotes osteogenic differentiation through FOXC1 upregulation in human bone mesenchymal stem cells [18]. Eskildsen et al. showed that the overexpression of miR-138 inhibits osteogenic differentiation of human mesenchymal stem cells [17]. Our present study is the first to suggest a suppressive role of miR-138 in the osteogenic differentiation of hDPSCs following TNF-*α* treatment. Our results may provide a new method for treating periodontal bone. However, the inflammatory environment in the dental pulp is complex. The role of miR-138 in osteogenic differentiation of hDPSCs in an inflammatory environment should be confirmed in animal models.

MicroRNAs play a role in suppressing the protein levels of their target genes. Therefore, the identification of the target gene is necessary to elucidate the regulatory mechanism of miR-138. Previous studies have revealed that FAK is a direct target of miR-138. Overexpression of miR-138 inhibits the osteogenic differentiation of hMSCs by repressing FAK expression, thus suppressing the FAK-ERK1/2 signaling pathway [17]. We found that the inhibition of miR-138 resulted in the upregulation of FAK, p-ERK1/2, and RUNX2 at the protein level, whereas the overexpression of miR-138 downregulated these proteins during the induction of osteogenic differentiation of hDPSCs under TNF-*α* treatment. These results indicate that miR-138 may regulate osteogenic differentiation of hDPSCs under TNF-*α* treatment by activating the FAK-ERK1/2 signaling pathway.

In summary, treatment with 50 ng/mL TNF-*α* induced miR-138 expression during the induction of osteogenic differentiation in hDPSCs, while miR-138 inhibition promoted the osteogenic differentiation of hDPSCs after treatment with 50 ng/mL TNF-*α*. miR-138 may be a key regulator of the osteogenic differentiation of hDPSCs following TNF-*α* treatment. However, the detailed mechanism and clinical application of miR-138 require further study. In addition to TNF-*α*, IFN-*γ* is also a major and potent proinflammatory cytokine that strongly inhibits osteogenic differentiation [31]. We did not discuss whether miR-138 has any effect on IFN-*γ*-induced suppression of osteogenic differentiation. This is a limitation of this study. In a future study, we will focus on this issue.

## Data Availability

All data generated and analyzed during this study are presented in the manuscript.
